# Supplementary material for: Causal Relationship between Aging and Anorexia Nervosa: A White-Matter-Microstructure-Mediated Mendelian Randomization Analysis
Source: Biomedicines. 2024 Aug 16;12(8):1874. doi: 10.3390/biomedicines12081874 (PMC11351342; doi:10.3390/biomedicines12081874)
Supplement: Supplementary file 1 [file biomedicines-12-01874-s001.zip › Supplementary Figure/Figure S4/Singe SNP plot of MR analysis for TL to ubm-b-2059.pdf]

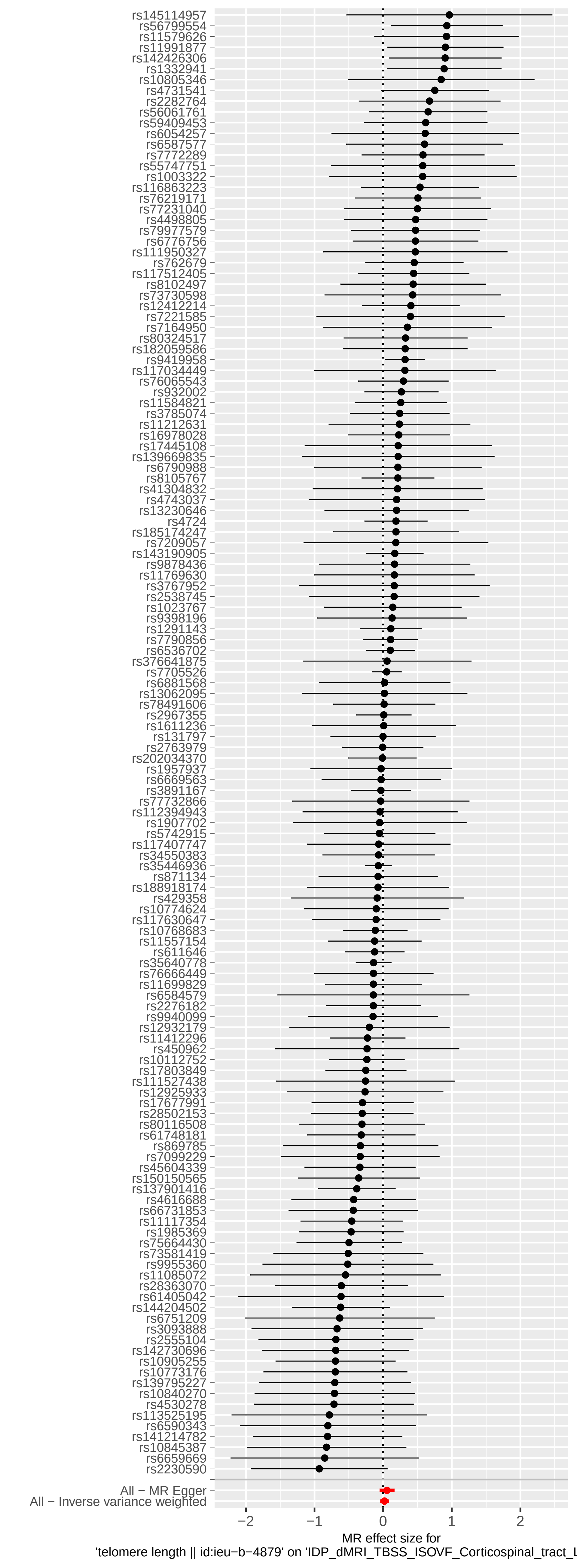
rs145114957
rs56799554
rs11579626
rs11991877
rs142426306
rs1332941
rs10805346
rs4731541
rs2282764
rs56061761
rs59409453
rs6054257
rs6587577
rs7772289
rs55747751
rs1003322
rs116863223
rs76219171
rs77231040
rs4498805
rs79977579
rs6776756
rs111950327
rs762679
rs117512405
rs8102497
rs73730598
rs12412214
rs7221585
rs7164950
rs80324517
rs182059586
rs9419958
rs117034449
rs76065543
rs932002
rs11584821
rs3785074
rs11212631
rs16978028
rs17445108
rs139669835
rs6790988
rs8105767
rs41304832
rs4743037
rs13230646
rs4724
rs185174247
rs7209057
rs143190905
rs9878436
rs11769630
rs3767952
rs2538745
rs1023767
rs9398196
rs1291143
rs7790856
rs6536702
rs376641875
rs7705526
rs6881568
rs13062095
rs78491606
rs2967355
rs1611236
rs131797
rs2763979
rs202034370
rs1957937
rs6669563
rs3891167
rs77732866
rs112394943
rs1907702
rs5742915
rs117407747
rs34550383
rs35446936
rs871134
rs188918174
rs429358
rs10774624
rs117630647
rs10768683
rs11557154
rs611646
rs35640778
rs76666449
rs11699829
rs6584579
rs2276182
rs9940099
rs12932179
rs11412296
rs450962
rs10112752
rs17803849
rs111527438
rs12925933
rs17677991
rs28502153
rs80116508
rs61748181
rs869785
rs7099229
rs45604339
rs150150565
rs137901416
rs4616688
rs66731853
rs11117354
rs1985369
rs75664430
rs73581419
rs9955360
rs11085072
rs28363070
rs61405042
rs144204502
rs6751209
rs3093888
rs2555104
rs142730696
rs10905255
rs10773176
rs139795227
rs10840270
rs4530278
rs113525195
rs6590343
rs141214782
rs10845387
rs6659669
rs2230590
All − MR Egger
All − Inverse variance weighted
−2
−1
0
1
2
MR effect size for
'telomere length || id:ieu−b−4879' on 'IDP_dMRI_TBSS_ISOVF_Corticospinal_tract_L
